# Current News

**Published:** 1898-06

**Authors:** 


### Current News.

### THE NATIONAL DENTAL ASSOCIATION.

THE next annual meeting of the National Dental Association will be held in Omaha, commencing on Tuesday, the 30th day of August, 1898.

Attention is called to the fact that all who were members of the American Dental Association and of the Southern Dental Association at the time of the formation of the National Dental Association are now members of the latter organization.

The Constitution, Article III., Section 5, provides as follows:

"It is hereby specially provided that all persons at present permanent members of the American Dental Association and of the Southern Dental Association are permanent members of this Association, and entitled to all the privileges of the class to which they belonged without further action, and the treasurer is hereby directed to transcribe their names upon the roll of membership of this Association."

The officers of the National Dental Association will leave nothing undone to make the meeting at Omaha a success, and they hope the attendance and interest in the first active annual meeting of the Association will be commensurate with its importance.

By order of

THOMAS FILLEBROWN,
*President.*
EMMA EAMES CHASE,
*Corresponding Secretary.*

### AMERICAN MEDICAL ASSOCIATION, SECTION ON STOMATOLOGY.

PROGRAMME of the Section on Stomatology of the American Medical Association at Denver, Col., June 7 to 10, 1898.

Dr. G. V. I. Brown, Milwaukee, Wis., Chairman's Address.

Dr. W. B. Hill, Milwaukee, Wis., "Methods of Teaching Materia Medica with Special Reference to the Needs of Dental Students."

Dr. G. T. Carpenter, Chicago, Ill., "A Method of Handling Alveolar Pyorrhœa."

Dr. W. H. Hall, Denver, Col., "Only a Baby Tooth."

Dr. J. Taft, Cincinnati, Ohio, "The Management of Pulpless Teeth."

Dr. L. G. Noel, Nashville, Tenn.

Dr. W. Knight, Cincinnati, Ohio, "Epulides."

Dr. V. A. Latham, Chicago, Ill., "Dental Septicæmia of the Antrum."

Dr. A. E. Baldwin, Chicago, Ill., "Some Facial Deformities and their Prevention."

Dr. A. H. Thompson, Topeka, Kan., "Jaw Movements in Relation to Tooth Forms."

Dr. Eugene S. Talbot, Chicago, Ill., "Irregularities of the Dental Arch."

Dr. V. A. Gudex, Milwaukee, Wis., "The Resistance of the Mucous Membranes to Bacterial Infection."

Dr. A. H. Sawins, Denver, Col., "The Morbid Susceptibility of Dental Structures is Greater than that of other Tissues."

Dr. J. M. Porter, Denver, Col., "Have We Progressed?"

G. V. I. BROWN, Milwaukee, Wis.,
*Chairman.*

EUGENE S. TALBOT, Chicago, Ill.,
*Secretary.*

---

### NEW JERSEY STATE DENTAL SOCIETY.

THE Twenty-eighth Annual Session of the New Jersey State Dental Society will be held at Asbury Park, commencing July 20, and continuing the 21st and 22d.

The commodious and pleasant "Auditorium" has been secured for the sessions, with unlimited space for clinics and exhibits.

Papers and clinics from many eminent dentists have already been secured.

Preparations are being made for the largest display of electrical exhibits of appliances for the use of dentistry ever before given, one-hundred-and-ten- and five-hundred-volt current being attainable.

The hotel Columbia adjoining has been secured for head-quarters for members and visiting friends; rates will be $2.50 and $3.00 per day.

CHARLES A. MEEKER, D.D.S.,
*Secretary.*
H. S. SUTPHEN, D.D.S.,
*Assistant Secretary.*

---

### NEW JERSEY SUMMER EXAMINATIONS.

THE New Jersey Dental Examining Board will hold the summer examinations at the commission rooms, No. 88 Broad Street, Elizabeth, N. J., commencing on Tuesday, July 5, and lasting for three days. All applications must be in the hands of the Secretary before June 21.

Attention is called to the new dental law of the State, which requires preliminary education equal to a high-school graduation in New Jersey. Candidates, who have not the necessary papers to show this standard will be given an examination held under the supervision of the State Superintendent of Public Instruction; all preliminary examinations must be finished before the applications can be accepted.

G. CARLETON BROWN,
*Secretary.*

NO. 88 BROAD STREET, ELIZABETH, N. J.

---

### MISSOURI STATE DENTAL ASSOCIATION.

THE Thirty-fourth Annual Meeting of the Missouri State Dental Association will convene at Merrimac Highlands (near St. Louis), July 5, 6, 7, and 8, 1898.

All dentists practising in the State who are not members and wish to become members of the Association and dentists of other States are cordially invited to attend.

When purchasing your railroad ticket, remember to get your certificate from the ticket agent, so that you can get the rebate on your return ticket.

A large attendance is anticipated, and we are assured of a royal meeting.

H. H. SULLIVAN,
*Recording Secretary.*

KANSAS CITY, MO.

---

### COLORADO STATE DENTAL ASSOCIATION.

THE Twelfth Annual Meeting of the Colorado State Dental Association will be held in Denver, June 7, 8, 9, and 10, in conjunction with the Stomatological section of the American Medical Association.

An excellent programme is assured. Those attending may avail themselves of reduced railroad rates. A cordial invitation is extended to all members of the profession.

ARTHUR C. WATSON,
*Chairman Executive Committee.*

---

### PENNSYLVANIA STATE DENTAL SOCIETY.

THE Executive Committee desires to announce that, on account of circumstances over which it had no control, the place of holding the annual meeting has been changed from Cresson to Ebensburg.

The Committee feels that in selecting the latter place it has acted wisely. Ebensburg is the county seat of Cambria County, and is a most delightful old town, located on the summit of the Alleghany Mountains, and readily accessible *via* Pennsylvania Railroad and Cresson. Head-quarters will be at the Maple Park Springs Hotel, and the meetings will be held in the court-house. Booklet and programme will be sent out about June 10.

Annual Session, July 12, 13, and 14.

I. N. BROOMELL,
*Chairman Executive Committee.*

